# Exploring the Effect of Relaxation Time, Natural Surfactant, and Potential Determining Ions (Ca^2+^, Mg^2+^, and SO_4_^2−^) on the Dynamic Interfacial Tension Behavior of Model Oil-Brine Systems

**DOI:** 10.1016/j.heliyon.2024.e29247

**Published:** 2024-04-04

**Authors:** Amir Mohammadi, Mahsa Parhizgar Keradeh

**Affiliations:** Faculty of Petroleum and Natural Gas Engineering, Sahand University of Technology, Tabriz, PO. Box: 51335-1996, Iran

**Keywords:** Dynamic interfacial tension, Salinity, Divalent ions, Heptol, Asphaltene, Relaxation time

## Abstract

This study examined how the concentration of asphaltene and divalent ions in various salinities affects the interfacial tension (IFT) between a model oil/brine using the pendant drop method. The oleic phase consisted of a mixture of toluene and n-heptane (heptol), to which asphaltene was added to investigate how asphaltene molecules affect the surface properties. The base fluid was prepared with a salinity of 40,000 ppm, and two additional solutions with concentrations of 4000 ppm (low salinity) and 80,000 ppm (high salinity) were created. The results revealed that increasing the concentration of asphaltene within certain salinity ranges led to a decrease in IFT. The lowest IFT was observed at the 40,000 ppm salinity level, indicating that at this optimal salinity, the maximum asphaltene concentration migrated to the heptol/brine interface, reducing the IFT from 23 mN/m to 16 mN/m. Additionally, a 0.5 % wt of asphaltene demonstrated a significant concentration of micellization of natural surfactants, suggesting that the interface was nearly saturated with asphaltene. Consequently, concentrations higher than this value did not significantly alter the IFT. In the final part of the study, the impact of divalent ions was investigated, revealing that as the concentration of Ca^2+^ ions increased up to fourfold, the IFT decreased to 15 mN/m, about 10 % less than the base case. This value represented the lowest IFT compared to Mg^2+^ and SO_4_^2−^. Moreover, modeling the results indicated that the relaxation time decreased with increasing salinity, suggesting that higher salinity accelerated the process of asphaltene absorption at the interface.

## 1 Introduction

Asphaltene is regarded as a natural surfactant inherently present within crude oil [[1], [2], [3], [4], [5], [6]]. Within the spectrum of crude oil components, asphaltenes and resins are acknowledged as surface-active agents, with asphaltene serving as the major polar component and surface-active agent in crude oil [[7], [8], [9], [10]]. Asphaltenes are specifically characterized as components that exhibit insolubility in normal alkanes but demonstrate solubility in aromatic solvents [11,12]. Over the past decade, there has been a growing focus on investigating the surface characteristics of asphaltenes at the interface between crude oil and brine in the oil industry, due to their significant role in surface phenomena. One of the techniques employed to gain insights into asphaltene adsorption at the crude oil/brine interface is the measurement of interfacial tension (IFT). Typically, the impact of asphaltenes on surface properties is examined by extracting them from crude oil and dissolving them in a blend of aromatic and aliphatic solvents, such as the commonly used mixture of normal heptane and toluene, often referred to as 'heptol' [7,10,12]. Numerous research attempts have explored the influence of various factors on the IFT between oil and brine. To develop a comprehensive understanding of this subject, it is essential to review a variety of studies.

Akstinat et al. (1981) conducted research indicating that the impact of temperature on IFT is significantly modulated by the constituents present in crude oil. In crude oil rich in naphthenic components, raising the temperature resulted in a decrease in IFT. On the other hand, the presence of paraffin and aromatic components does not exert any influence on IFT [13]. Yarranton et al. (2005) observed that the concentration of asphaltenes has an impact on the interfacial characteristics between oil and brine. Their investigation focused on a system composed of toluene/asphaltene/brine. Their findings indicated that the oil phase containing a higher asphaltene concentration tends to produce a less stable emulsion compared to the oil phase with a lower asphaltene concentration [3]. Lashkarbolooki et al. (2014) examined how various salts presenting in the aqueous phase, influence the IFT of acidic oil and brine. Their findings revealed that divalent cations like Mg^2+^ and Ca^2+^ exert a more pronounced impact on IFT compared to other ions. Additionally, they noted that NaCl and KCl result in the highest levels of IFT [14]. Hu et al. (2016) conducted a study examining the surface characteristics of asphaltenes at the interface between heptol/brine. They explored different asphaltene concentrations ranging from 0.001 % to 1 % wt, as well as various heptol ratios. Their findings revealed that under constant salinity conditions, the IFT of the toluene/heptol mix (75 % toluene and 25 % heptane), 50/50 and 75/25 progressively decreased over approximately 15 h as the asphaltene concentration increased. In contrast, in the case of the 25/75 heptol mixture, the IFT initially decreased for about 1.5 h but then increased, indicating the asphaltene precipitation [7]. In their 2016 study, Khaksar Manshad et al. examined the impact of water-soluble ions on the IFT between crude oil/brine. Their findings revealed that as the concentration of each ion is raised within a specific optimal range, the IFT decreases. However, beyond this optimal range, when the concentration is increased further, the IFT begins to rise. Additionally, they observed that the optimal concentration varies across different systems [15]. In another study carried out by Mohammadi et al. (2020), the dynamic pendant drop method was employed to investigate adsorption kinetics at the oil/brine interface. Various heptol ratios represented crude oil models. Results revealed that higher asphaltene concentrations reduced IFT, particularly with increased n-heptane fractions. The Ward-Tordai short-time model estimated asphaltene diffusion coefficients for dilute solutions in heptol. Findings suggested asphaltene adsorption as monomers at the interface, with n-heptane promoting faster adsorption, lower IFT, and enhanced emulsification [10]. Ashoorian et al. (2023) explored the challenges in predicting and controlling asphaltene behavior in the oil and gas industry. Through dynamic light scattering (DLS), dynamic IFT, and interfacial rheology measurements, the impact of bulk aromaticity on asphaltene surface behavior was investigated. Low aromaticity conditions increased asphaltene surface activity, altering dilational rheological parameters. For instance, in heptol 50/50, instantaneous elasticity was approximately 40 mN/m (around 0.08 Hz), contrasting with toluene, where it decreased to about 30 mN/m at higher frequencies (about 0.12 Hz). The study emphasized the importance of aging time, highlighted the significance of dynamic interfacial data over static data, and suggested a direct correlation between surface activity and solution aromaticity [16].

Previous research has established that the presence of polar compounds in crude oil, such as asphaltene, and different types of ions in the aqueous phase, have a noteworthy impact on interfacial tension. In light of the significance of this subject, in this paper, the Mono Exponential Decay Model was employed to model the relaxation time, determining how long it takes for asphaltene to reach the oil/brine interface, as well as this, the effect of divalent ions was investigated. To ensure that the influence of other components in crude oil remained constant, a model oil, known as heptol comprising equal parts toluene and normal heptane (50 % n-heptane and 50 % toluene), was employed. This allowed for the examination of how changes in asphaltene concentration affect IFT. Subsequently, asphaltene was added to heptol, and the impact of varying asphaltene concentration and divalent ions including Ca^2+^, Mg^2+^, and SO_4_^2−^ in aqueous phase on the IFT of heptol/brine was investigated across a broad salinity range. The dynamic IFT was measured using the pendant drop method, and the resulting data were subjected to mathematical modeling. This research outcome gains a deep insight into the surface phenomenon, which is essential for enhancing oil recovery.

## 2 Experimental section

### 2.1 Brine

In this study, the brine employed for the experiments was prepared by dissolving various salts (with a purity exceeding 99 %) in distilled water as outlined in Table 1. The research used a reference fluid with a salinity of 40,000 ppm as a base case, and also examined the impact of low salinity (4000 ppm) and high salinity (80,000 ppm) water to explore salinity-related effects [17].

**Table 1:** The composition of different brines (in ppm) that are used in this study.

Ion	4000 ppm	40,000 ppm	80,000 ppm
Na^+^	1724	17243	34486
Cl^−^	1981	19807	39614
K^+^	40	400	800
HCO^3-^	5	50	100
Mg^2+^	214	2143	4286
Ca^2+^	46	460	920
SO_4_^2-^	150	1497	2994
TDS	4162	41659	83318
Ionic strength, mol/L	0.083	0.832	1.664

### 2.2 Heptol

A model oil was prepared by blending toluene and regular heptane in equal proportions (in a 1:1 vol ratio). The asphaltene used in this mixture was derived from crude oil sourced from a southern Iranian reservoir using the IP-143 standard extraction method. Initially, normal heptane was added to the asphaltene in a quantity 40 times the volume of the oil. The mixture was then stirred with a magnetic stirrer for a duration of 4–5 h. Subsequently, the resulting dark solution was allowed to settle in ambient conditions for 24 h to separate any undissolved particles. During this stage, the dark solution was filtered through 1–1.5 μm filter paper and left undisturbed for another 24 h. In the final step, asphaltene extraction was carried out by placing the sample in a soxhlet apparatus, and the washing process with normal heptane was continued until the heptane turned clear [9,12,18].

### 2.3 IFT measurement apparatus and procedure

**Fig. 1:**
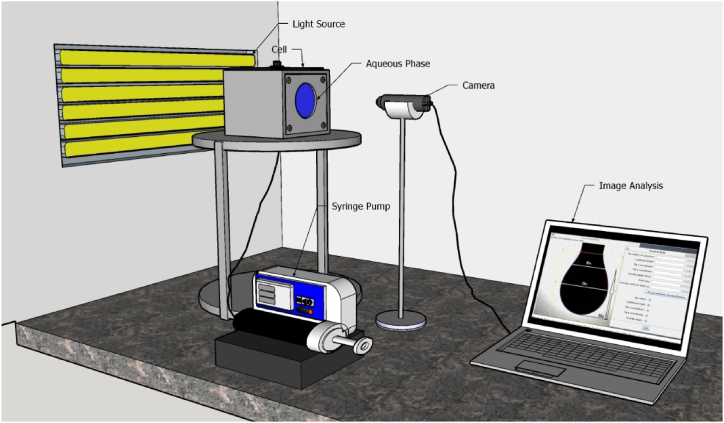
The schematic of IFT apparatus.

Fundamental investigations were conducted to assess the IFT in the Heptol/brine system. This was achieved through the application of the pendant drop technique, performed at atmospheric pressure and a temperature of 30 °C. Fig. 1 provides an illustration of the apparatus employed for IFT measurement using the pendant drop method. In this approach, a constant-volume Heptol droplet was introduced into a chamber containing the brine (aqueous phase) using a syringe pump. Through real-time image processing, the droplet's profile was continuously monitored until it reached an equilibrium state. Subsequently, the IFT was calculated using equation (1).

(1) $$\gamma =\frac{\Delta \rho gD^{2}}{H}$$

In this context, γ represents the interfacial tension, Δρ denotes the disparity in density between the oleic and aqueous phases (measured in g/cm³). The variable 'g' stands for gravitational acceleration (in cm/sec^2^), and 'D' (or 'd') refers to the larger (or smaller) diameter of the droplet (in cm). The parameter 'H' serves as an adjustment factor, which is associated with the shape factor denoted as 'S' and calculated as 'd/D'.

**Fig. 2:**
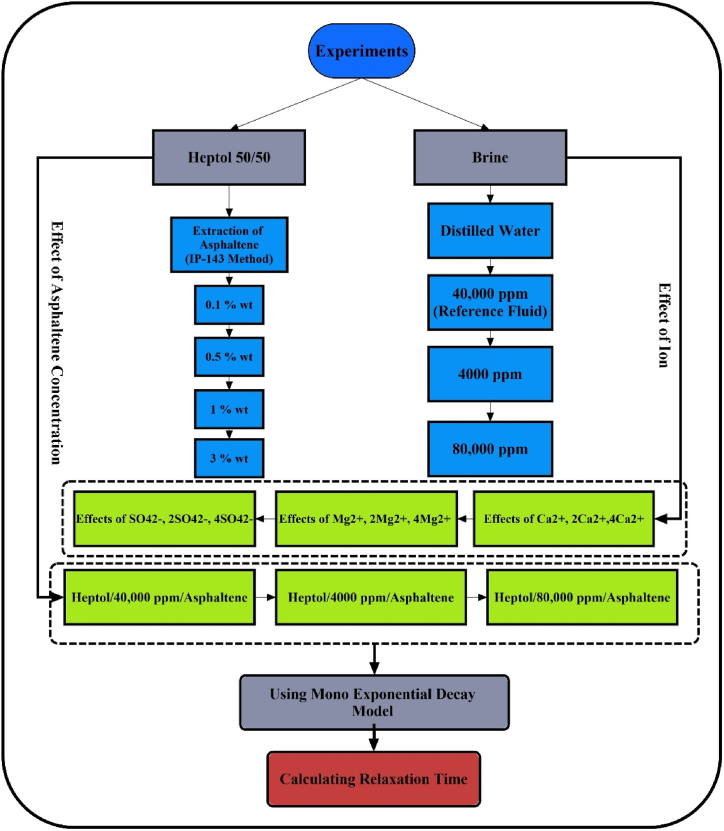
Flowchart of materials and array of tests.

Initially, the apparatus underwent calibration using distilled water/n-heptane, as well as distilled water/toluene, at a temperature of 30 °C and standard atmospheric pressure. The device's precision stands at approximately ±0.3 mN/m. The procedure for measuring IFT is a dynamic one, and data were recorded as a function of time until reaching equilibrium. It's worth noting that the time required to attain equilibrium in IFT tests was nearly consistent across all samples. To enhance the accuracy of IFT measurements and verify result reproducibility, each test was conducted a minimum of twice. Fig. 2 illustrates the experimental flowchart along with the array of tests.

## 3 Results and discussion

### 3.1 The effect of asphaltene concentration on the IFT of the heptol/brine system

**Fig. 3:**
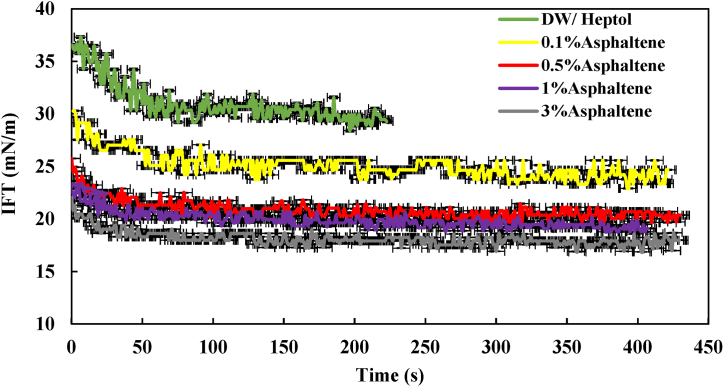
IFT profile of the heptol/distilled water system at various asphaltene concentrations.

In this section, we examined the impact of asphaltene concentrations at 0.1 %, 0.5 %, 1 %, and 3 % wt on the IFT of the Heptol/brine system. Initially, we investigated the influence of asphaltene on the IFT within the heptol/distilled water system. Fig. 3 illustrates the IFT profile of the Heptol/distilled water system at various asphaltene concentrations.

As depicted in the figure, the highest IFT value was observed for the Heptol solution without asphaltene. After approximately 75 s, the IFT reduced from 36.55 mN/m to 29.45 mN/m. In general, it can be noted that in the absence of surface-active agents, the IFT tends to be higher. Introducing 0.1 % wt of asphaltene into the solution led to a significant reduction in IFT, reaching an equilibrium value of 23.77 mN/m. This indicates that asphaltene molecules, with their hydrophilic group in the aqueous phase and hydrophobic group in the organic phase, tend to adsorb at the water-oil interface, lowering the IFT, similar to surfactants [8,19,20]. As the asphaltene concentration increased from 0.5% to 3 % wt, a similar trend of decreasing IFT was observed, the same as the effect seen with a 0.1 % concentration. It's noteworthy that the lowest IFT value for this system was achieved with 3 % wt of asphaltene, reaching 18 mN/m.

**Fig. 4:**
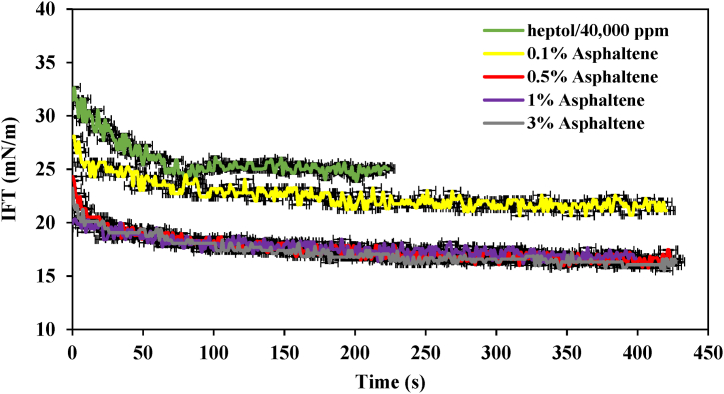
IFT profile of the heptol/40,000 ppm system at various asphaltene concentrations.

According to Fig. 4, which shows the IFT profile of the heptol/40,000 ppm system at various asphaltene concentrations, the highest IFT was observed in the sample lacking asphaltene. In this sample, within the initial 50 s, the IFT exhibited a rapid decrease, dropping from 33 mN/m to 25 mN/m after roughly 80 s, at which point it reached equilibrium. This reduction, in the absence of asphaltene molecules, can be attributed to the presence of ions, primarily divalent positive ions, found in the brine. Upon introducing 0.1 % wt of asphaltene, a notable reduction in IFT compared to the previous sample was observed. The IFT decreased from 28 mN/m to 21 mN/m. This decrease can be rooted in the simultaneous presence of a hydrocarbon skeleton with a hydrophobic structure and a polar group with a hydrophilic structure in asphaltene molecules. This characteristic makes them active molecules at the interface between brine and heptol [7,8,12,21]. As a result, the hydrophilic group resides in the aqueous phase, while the hydrophobic group is situated in the organic phase, leading to a reduction in IFT. Once the system reaches thermodynamic equilibrium, the IFT stabilizes [11,22,23].

Increasing the asphaltene concentration to 0.5 % wt resulted in a more pronounced reduction in the IFT profile. Specifically, within the first 10 s, the IFT for this sample dropped from its initial value of 24.8 mN/m to 20 mN/m. Towards the end, it reached an equilibrium value of 16.5 mN/m with a gentler slope. Generally, the results obtained at concentrations of 1% and 3% wt were similar to those at the 0.5 % wt, although the equilibrium IFT for 1 % and 3 % by weight concentrations were 16.6 mN/m and 16 mN/m, respectively. The results indicate that at a concentration of 0.5 % wt, the Heptol/brine interface contains the highest concentration of asphaltenes, and beyond this concentration, there is limited capacity for further asphaltene absorption at the interface. This phenomenon is reminiscent of the critical micelle concentration (CMC) for surfactants, where concentrations above the CMC result in micelle formation, and excess surfactant molecules tend to aggregate into micelles [9,12,24,25].

**Fig. 5:**
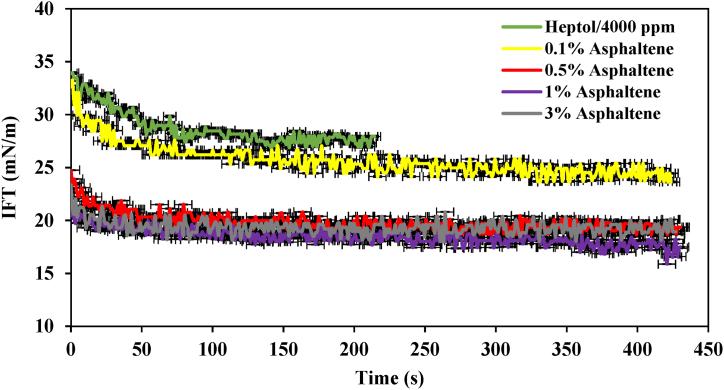
IFT profile of the heptol/4000 ppm system at various asphaltene concentrations.

Fig. 5 depicts the IFT profile of the Heptol/4000 ppm system at varying asphaltene concentrations. According to the figure, the initial IFT for the heptol/4000 ppm system without asphaltene was approximately 34 mN/m. This initial IFT was higher than that observed in the 40,000 ppm system without asphaltene. Over time, it exhibited a gradual decrease, reaching 29 mN/m after 50 s. These results underscore the beneficial influence of brine ions in reducing IFT. Upon introducing 0.1 % asphaltene into the system, a notable decrease in IFT occurred with a relatively steep slope. After approximately 50 s, the rate of decrease slowed, ultimately reaching an equilibrium value of 24.2 mN/m, as expected. With the increase in asphaltene concentration to 0.5 % and 1 % wt, a similar downward trend in equilibrium IFT was observed, reaching values of 19.3 mN/m and 17.5 mN/m, respectively. In the case of the 3 % wt, the trend was more or less similar to the 1 % wt, resulting in an equilibrium IFT of 18.9 mN/m. This behavior can be attributed to the CMC phenomenon, as previously mentioned.

**Fig. 6:**
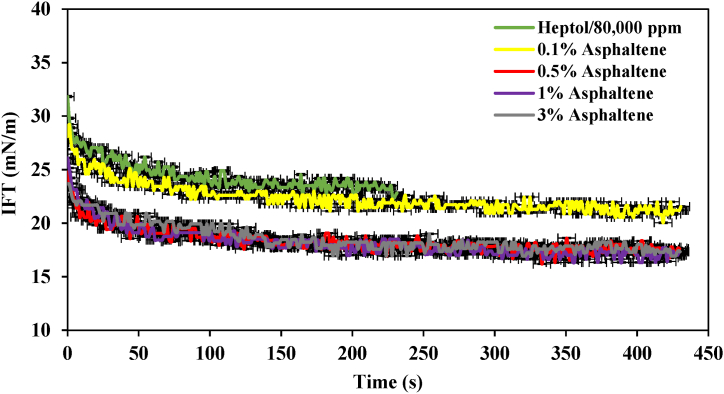
IFT profile of the heptol/80,000 ppm system at various asphaltene concentrations.

Subsequently, an investigation was conducted on the heptol/80,000 ppm system with varying asphaltene concentrations, as depicted in Fig. 6. The overall trend in IFT followed a pattern consistent with other salinity levels. Initially, there was a swift, minor decrease in IFT, followed by a more prolonged reduction, eventually reaching an equilibrium state. To elaborate, the sample lacking asphaltene exhibited the highest IFT, with the IFT decreasing from 32 mN/m to an equilibrium value of 24 mN/m. Upon introducing 0.1% asphaltene into the system, the IFT reached an equilibrium value of 21 mN/m. However, with higher asphaltene concentrations, specifically 0.5 %, 1 %, and 3 %, a significant reduction in IFT was observed. The most substantial decrease was observed in the 1 % asphaltene sample, where the IFT decreased from 22 mN/m to an equilibrium value of 17.3 mN/m.

**Fig. 7:**
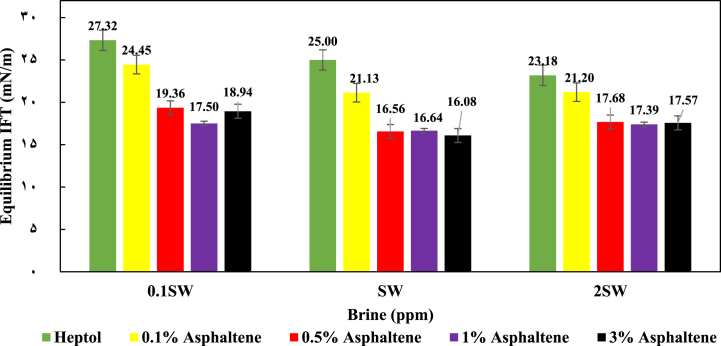
Equilibrium IFT of Heptol/brine system in different asphaltene concentrations and different salinities.

Fig. 7 presents a comparison of the equilibrium IFT under various salinity levels and asphaltene concentrations. In a general sense, it can be observed that both the concentration of asphaltene and the salinity within the examined system exhibit an optimal range. The findings indicate that when the asphaltene concentration was increased to 0.5 % wt, there was a significant decrease in IFT across all samples. However, when the asphaltene concentration was further raised to 1 % wt, the reduction in IFT was less pronounced compared to the 0.5 % wt. It can be inferred that the interface of heptol/brine is primarily saturated with asphaltene within the concentration range of 0.5 % wt to 1 % wt. Consequently, pushing the asphaltene concentration beyond this range does not yield further reduction in IFT. This suggests the presence of an optimal salinity range that facilitates the migration of asphaltene molecules to the interface of heptol/brine. Other comparable research has also identified the optimal salinity range. Specifically, Mohammadi et al.'s investigation reported that within the optimum salinity range, a higher concentration of asphaltene (1 %wt in their study) could effectively migrate to the oil/brine interface. The results indicated that at elevated salinity levels, the transportation of polar components to the interface could be accelerated [26]. This behavior may be analogous to the way commercial surfactants behave in systems involving diverse oils and brines. This behavior may be analogous to the way commercial surfactants behave in systems involving diverse oils and brines [11,24,[27], [28], [29]].

### 3.2 The effect of divalent ions on the IFT of heptol/brine system

**Fig. 8:**
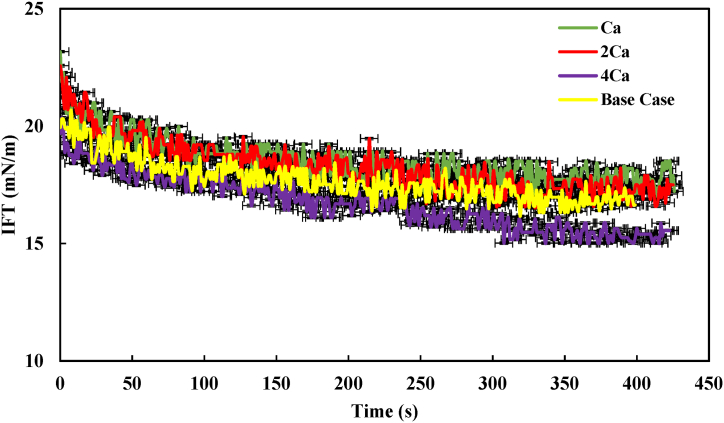
IFT profile of the heptol/40,000 ppm system (containing Ca^2+^) in the presence of 1 % asphaltene.

In this section, we examined how the presence of divalent ions dissolved in brine affects the IFT in the presence of 1 % asphaltene. In most of the samples, the IFT decreased until it reached a concentration of 1 % wt. Beyond this concentration, a reversal in the trend occurred, specifically in the samples with 4000 and 80,000 ppm. As a result, we selected a concentration of 1 % wt asphaltene for further investigation. These tests were conducted at a temperature of 30 °C. To elaborate, we maintained the ionic strength constant while only increasing the concentration of divalent ions (Ca^2+^, Mg^2+^, and SO_4_^2−^) up to four times for further analysis. These samples were then compared with the 40,000 ppm sample (as a base case), which contains all the ions typically found in brine, as indicated in Table 1. Fig. 8 displays the IFT of the Heptol/40,000 ppm system (containing Ca^2+^) in the presence of 1% asphaltene. The figure illustrates that as the concentration of Ca^2+^ in the system increased from one to four times, the IFT of the system initially rose and then decreased. At its lowest point (4Ca^2+^), the IFT reached approximately 15 mN/m.

**Fig. 9:**
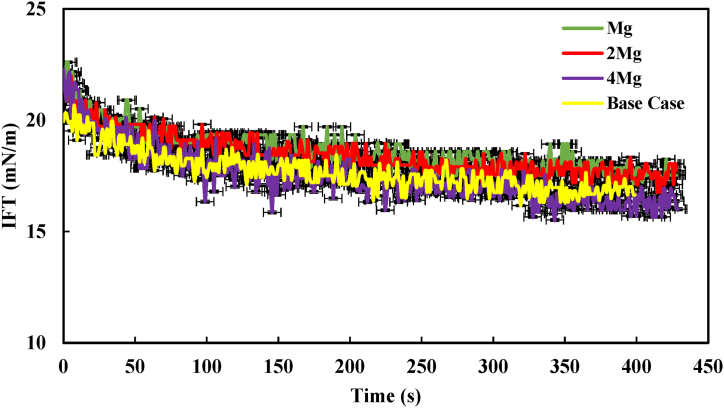
IFT profile of the heptol/40,000 ppm system (containing Mg^2+^) in the presence of 1 % asphaltene.

**Fig. 10:**
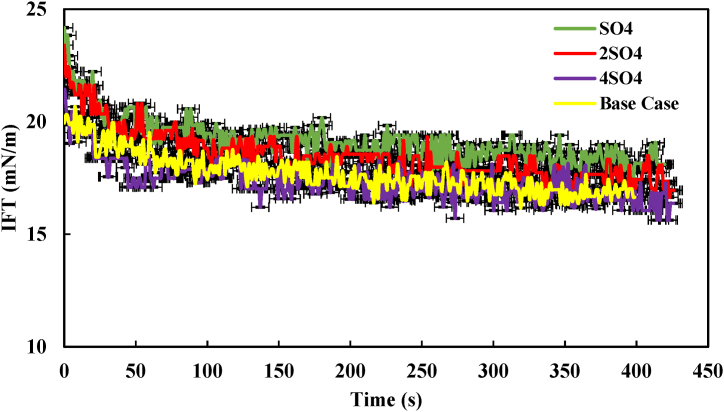
IFT profile of the heptol/40,000 ppm system (containing SO_4_^2−^) in the presence of 1 % asphaltene.

However, in Fig. 9, Fig. 10, which respectively depict the IFT of the heptol/40,000 ppm system in the presence of Mg^2+^ and SO_4_^2−^, a different pattern emerges. It was evident that as the concentration of Mg^2+^ and SO_4_^2−^ was increased up to four times, the IFT eventually approached the base case. This behavior could be attributed to the high salinity conditions and the presence of divalent ions, which led to the depletion of asphaltene at the interface, making it difficult for the IFT to decrease further [8,9,30,31]. It's worth noting that Ca^2+^, Mg^2+^, and SO_4_^2−^ in the aqueous phase exhibit a strong affinity for bonding with water molecules, resulting in hydration by disrupting these water molecule bonds. Conversely, the presence of polar compounds like asphaltene in the oil phase, encourages the accumulation of natural surfactants at the heptol/brine interface, leading to a reduction in IFT [12,19]. Previous research has also indicated that each ion dissolved in brine may have a distinct impact on the oil/water IFT. For instance, Ca^2+^ has been reported to have an affinity for interacting with asphaltene molecules. Consequently, the synergy between Ca^2+^ and asphaltene, as well as their presence at the interface, can lead to a more significant reduction in IFT compared to other ions [31]. Ultimately, the comparison of the divalent ions revealed that the increase in Ca^2+^ concentration up to four times resulted in a more substantial IFT reduction than Mg and SO_4_^2−^. The findings have been corroborated through additional investigations carried out by Mohammadi et al. Their study emphasized that among various divalent ions, Ca^2+^ exerted the most significant influence on the heptol/brine system [26].

### 3.3 Modeling the dynamic IFT of heptol/brine in the presence of asphaltene

Overall, the dynamic characteristics of IFT primarily stem from the existence of polar components within oil, such as asphaltene, and the presence of ions in the brine that migrate toward the brine-oil interface [28,32]. In these experiments, the IFT dynamically decreased as a consequence of asphaltene's presence in the oleic phase, gradually approaching a stable level. To analyze the outcomes, fine-tune the model to align with the laboratory data, and determine the relaxation time (absorption), the Mono Exponential Decay Model outlined in equation (2) was employed. The theoretical basis of this mono-exponential decay model lies in the assumption that the dynamic behavior of IFT in studied system can be adequately described by a single exponential decay process. This model is rooted in the idea that the IFT approaches its equilibrium value exponentially over time, with the rate of decay determined by the relaxation time (τ). The equation captures the transition from the initial IFT ($\gamma_{0}$) towards the equilibrium IFT ($\gamma_{eq}$). The mono-exponential decay model is commonly employed in various scientific disciplines when studying relaxation phenomena, and its use in the present paper is justified by its simplicity and ability to capture the essential features of the dynamic IFT behavior in model oil/brine system. However, it is important to acknowledge the limitations of this model. For instance, it assumes a single relaxation time for the entire process, which might oversimplify the complex interactions in some systems. Additionally, deviations from a pure mono-exponential behavior may occur in certain conditions [33,34].

(2) $$\gamma_{t}=\gamma_{eq}+{\left(\gamma_{0}-\gamma_{eq}\right)}^{e^{\left(-\frac{t}{\tau }\right)}}$$

Within this equation, $\gamma_{t}$ corresponds to the IFT at a specific time point t, $\gamma_{eq}$ denotes the IFT at equilibrium, $\gamma_{0}$ signifies the initial IFT, and τ serves as the parameter indicating the relaxation time.

Table 2 is presented, detailing the experimental conditions for the analysis of the Mono-Exponential Decay Model. Various mixtures were examined, encompassing a spectrum of asphaltene concentrations and salinities, allowing for a comprehensive exploration of dynamic IFT behavior. The model parameters, including relaxation time, initial IFT, and equilibrium IFT were tuned through an iterative process based on these experimental conditions. The values presented in the table represent a diverse array of scenarios investigated in this study, enabling a comprehensive understanding of the application of the model across different oil-brine mixtures.

**Table 2:** Different mixtures employed for mono-exponential decay model analysis.

Mixture	Asphaltene Concentration (% wt)	Salinity (ppm)
Mixture 1	0.1%	0
Mixture 2	0.5 %	0
Mixture 3	1 %	0
Mixture 4	3 %	0
Mixture 5	0.1%	4000
Mixture 6	0.5 %	4000
Mixture 7	1 %	4000
Mixture 8	3 %	4000
Mixture 9	0.1%	40000
Mixture 10	0.5 %	40000
Mixture 11	1 %	40000
Mixture 12	3 %	40000
Mixture 13	0.1%	80000
Mixture 14	0.5 %	80000
Mixture 15	1 %	80000
Mixture 16	3 %	80000

The relaxation time serves as a critical parameter, as it helps estimate the duration required for asphaltene to migrate to the interface between water and oil [24,35,36]. This estimation is crucial for determining how quickly polar compounds are absorbed in the oil/brine interface, ultimately reducing the IFT. By studying the relationship between the time, it takes for IFT to reach equilibrium and the initial IFT, we were able to calculate the absorption time for asphaltene. Using this model and examining the impact of factors like salinity and asphaltene concentration, it becomes feasible to pinpoint the minimum time needed for asphaltene to be absorbed at the interface. Although this model exhibited a satisfactory fit with the laboratory data, with the exception of the initial times, the discrepancy at those initial time points suggests that the relaxation time does not strictly follow an exponential pattern [2].

This study presents a comprehensive analysis of the Mono Exponential Decay Model's accuracy in predicting the dynamic IFT behavior of Heptol/brine systems under different asphaltene concentrations, as illustrated in Table 3. The model's performance is assessed using the coefficient of determination (R^2^) as a measure of accuracy, where R^2^ values fall between 0 and 1. Results for asphaltene concentrations of 0.1 %, 0.5 %, 1 %, and 3 %, across diverse brine compositions (4000 ppm, 40,000 ppm, and 80,000 ppm), are provided. The obtained R^2^ values demonstrate the model's reasonable accuracy in capturing the observed trends. This comparative analysis aims to highlight the reliability of the model across a spectrum of asphaltene concentrations.

**Table 3:** Comparative analysis of mono exponential decay model accuracy for varying systems.

Aqueous phase	0.1 %Asphaltene	0.5 % Asphaltene	1 % Asphaltene	3 % Asphaltene
Deionized water	0.6860	0.8466	0.7743	0.6586
4000 ppm	0.8274	0.7189	0.6514	0.5710
40,000 ppm	0.8276	0.8811	0.7964	0.9165
80,000 ppm	0.8706	0.8105	0.8504	0.8969

**Fig. 11:**
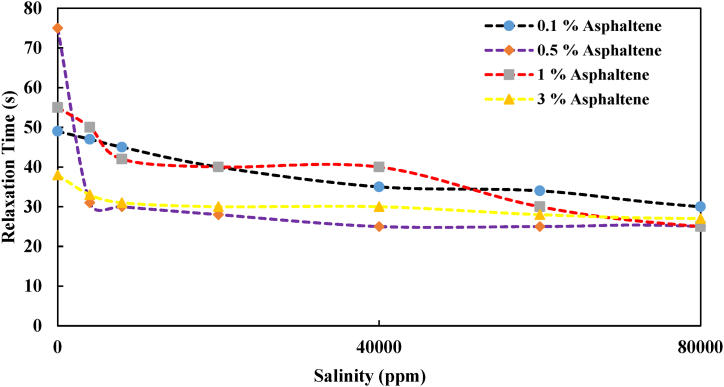
Relaxation time in heptol solutions with different concentrations of asphaltene and salinity range.

Fig. 11 illustrates the relaxation time in Heptol-based systems containing various asphaltene concentrations (0.1 %, 0.5 %, 1 %, and 3 %) across different salinity levels. Notably, in the model oil sample with 0.1 % asphaltene concentration, the relaxation time gradually decreased, moving from 50 s to 30 s. When asphaltene was introduced into the system at a 0.5 % concentration and salinity was increased to 4000 ppm, the relaxation time, along with the asphaltene absorption time at the interface, significantly decreased to 30 s. This effect appears to be influenced by the presence of various ions in the brine. Additionally, as salinity increased, the relaxation time decreased at a milder rate. In the sample with 1 % asphaltene, the general trend indicated a reduction, with the relaxation time decreasing from 55 s to 25 s. In the 3 % asphaltene sample, while the decreasing trend in asphaltene absorption time and relaxation time persisted, it had a gentler slope compared to other samples. Therefore, it can be inferred that increasing salinity accelerates the process of asphaltene absorption at the interface [37]. Furthermore, in the low asphaltene concentration (0.1 % wt), the relaxation time was longer compared to other concentrations in the studied system.

It is crucial to note that the dynamic characteristics of the IFT between crude oil and water are primarily influenced by the polar components or surfactants present in the fluids. This model can be extended to diverse mixtures. Previous research indicates that the Mono Exponential Decay Model has demonstrated satisfactory performance across various oil types, including acidic and non-acidic (basic) crude oils [38,39]. Specifically, Mohammadi et al. conducted a similar study that yielded excellent results. Their research involved extracting asphaltene from heavy crude oil. In contrast, our study obtained asphaltene from light crude oil using the IP-143 method outlined in the preceding section [26]. Furthermore, the model has shown promising results across different salinity ranges (from deionized water to formation water), temperatures (up to 80 °C), and pressures (up to 4000 psi) [40,41].

In summary, the results from this section suggest that, in general, higher salinity and greater asphaltene concentration in the system reduce the time required for asphaltene to be absorbed at the interface. However, it's worth noting that there appears to be an optimal value, beyond which increasing salinity (from 40,000 ppm to 80,000 ppm) doesn't significantly decrease asphaltene absorption time at the interface. This indicates that a synergistic effect between salinity and asphaltene concentration contributes to a more rapid reduction in dynamic IFT.

## 4 Conclusion

The research was conducted with the aim of investigating the surface behavior of asphaltene and assessing the effect of divalent on the oil/brine interface. The results indicated that the presence of asphaltene molecules, up to a concentration of 0.5 %, caused a noticeable decrease in IFT across all salinity ranges. For example, in the case of a 0.5 % wt asphaltene concentration, the IFT decreased by 29 %, 33 %, and 24 % compared to the absence of asphaltene in the system in 4000 ppm, 40,000 ppm, and 80,000 ppm, respectively. However, with an increase in asphaltene concentration to 1 % and 3 % wt, the IFT did not change significantly. For instance, at 4000 ppm, only a 2 mN/m decrease was observed. This phenomenon can be ascribed to the salinity range that optimally facilitates the migration of asphaltene molecules to the interface. In the subsequent section, an examination of the impact of divalent ions, such as Ca^2+^, Mg^2+^, and SO_4_^2−^, revealed that when 4Ca^2+^ was present in a 40,000 ppm solution, the IFT reached 15 mN/m, representing a decrease of about 10 % compared to the base case, and this was the lowest value among all the samples. Additionally, through data modeling, it was determined that the relaxation time decreases as salinity increases, indicating that higher salinity accelerates the process of asphaltene absorption at the interface. Furthermore, the findings in this article suggested that increasing salinity and optimizing asphaltene concentration could result in a more rapid reduction of dynamic IFT.

## Data availability statement

Data included in article/supp. material/referenced in article.

## CRediT authorship contribution statement

**Amir Mohammadi:** Writing – original draft, Formal analysis, Data curation, Conceptualization. **Mahsa Parhizgar Keradeh:** Writing – review & editing, Visualization, Validation.

## Declaration of competing interest

The authors declare that they have no known competing financial interests or personal relationships that could have appeared to influence the work reported in this paper.
